# Functional Roles for Exosomal MicroRNAs in the Tumour Microenvironment

**DOI:** 10.1016/j.csbj.2016.10.005

**Published:** 2016-10-27

**Authors:** Emma Bell, Molly A. Taylor

**Affiliations:** a AstraZeneca, Personalised Healthcare and Biomarkers, iMed, Cambridge Science Park, Cambridge CB4 0FZ, United Kingdom; b AstraZeneca, Oncology iMed Bioscience, Cambridge CB2 0RE, United Kingdom

**Keywords:** MicroRNA, Extracellular vesicles, Tumour microenvironment

## Abstract

Extracellular microRNAs are released from cells both passively and actively. The presence of these microRNAs in the tumour microenvironment (TME) can significantly impact on the plasticity of cancer cells leading to the promotion of metastatic and angiogenic processes. These extracellular microRNAs can act not only on other cancer cells, but also cells present in the TME, such as immune cells, endothelial cells, fibroblasts, and others acting to subvert the host immune system and drive tumour progression. In this review we highlight the current understanding of both the mechanisms by which microRNAs are released from tumour cells and the downstream functional effects that extracellular microRNAs have on recipient cells.

## 1 Introduction

Cancer research has traditionally focused on tumour intra-cellular gene expression and signalling pathway activation. This view postulates that cancer cells proliferate due to genetic mutations that activate growth signalling pathways. While these mechanisms are necessary and important events in tumour initiation and progression, they do not account for the complexity of the microenvironment in which the tumour sits. Tumours are complex systems composed of not only tumour cells, but stroma containing blood and lymph vessels, fibroblasts, endothelial cells, and immune cells among others [1]. This TME plays an essential role in the initiation, growth, and metastatic spread of cancer. Additionally, while genetic mutations initiate tumourigenesis, numerous post-transcriptional and post-translational mechanisms are at play within tumour cells and other cells within the TME that ultimately contribute to tumour progression. A large proportion of the human genome is made up of non-coding RNAs, including two of the most well studied classes of non-coding RNAs; long noncoding RNA (lncRNA) which are ~ 200 or more nucleotides in length and microRNAs, which are small (17–27 nucleotide) non-coding RNAs that regulate approximately 30–60% of all protein-coding genes through post-transcriptional mechanisms [2], [3]. MicroRNAs regulate gene expression by binding to the 3′UTR of target mRNAs, resulting in translation repression or RNA degradation [4]. Approximately 50% of microRNAs are located in regions of chromosomal abnormalities that are associated with cancer [5], meaning that in cancer cells with genetic abnormalities significant changes in specific microRNA clusters are likely. Particular microRNAs are known to act as both tumour suppressors and oncogenes in the development of tumours. For example, the miR-17-92 family of microRNAs is one of the most well characterised oncomiRs and has been shown to exert anti-apoptotic effects through its ability to downregulate Bim and PTEN tumour suppressors [6]. MiR-21 and miR-155 are also well characterised oncomiRs that promote both tumour growth and metastasis by targeting numerous mRNAs. In contrast, miR-15a, miR-16-1, miR-34a, and the let-7 family of microRNAs have been shown to suppress tumour growth and metastasis by inducing apoptosis, cell cycle arrest and senescence (reviewed in [4]). In addition to their functional effects on tumour cell signalling pathways, microRNAs have been shown to exhibit tissue specific expression patterns [7], suggesting that they have potential utility as clinical biomarkers [8].

Cell-free microRNAs are found in the circulation and since their discovery have become promising diagnostic, prognostic, and therapeutic response biomarkers for cancer. Indeed, circulating microRNA profiles can be used to identify disease types, with elevated circulating microRNAs being significantly associated with disease-associated genetic variants [9]. These circulating microRNAs have been found to be present in several different body fluid types [10], [11] and are incredibly stable, being able to withstand room temperature for extended periods of time and numerous freeze–thaw cycles [11]. The stability of endogenous microRNAs is in direct contrast to synthetic exogenous microRNAs spike-ins (e.g. cel-miR-39-3p) which when added to serum or media are rapidly degraded [11], [12]. Based on this evidence, circulating microRNAs have been hypothesised to be protected by being enclosed in extracellular vesicles or bound to proteins [11], [12], [13]. Given their prevalence and stability in biofluids it is not surprising that recent evidence points to extracellular microRNAs playing functional roles as autocrine, paracrine, and endocrine signalling molecules.

Here we review the mechanisms governing cellular release of microRNAs and the evidence for extracellular microRNA activity in cell-cell communication with a focus on how cell-free microRNAs have been shown to have functional roles influencing tumour progression and metastasis.

## 2 Extracellular Vesicles

The term extracellular vesicle encompasses exosomes, microsomes and apoptotic bodies [14], [15]. Exosomes are 30–100 nm in size and are formed from inward budding of the endosomal membrane [16]. This forms a multivesicular body (MVB) which contains several exosomes. The MVB then fuses with the plasma membrane and releases the exosomes from the cell [17], [18]. The resulting vesicles contain cytosol and can be characterised by the presence of tetraspanin proteins CD9, CD63, CD81, and CD82 [15]. Other vesicles in the circulation include microsomes which are produced from the disruption of the plasma membrane and are of a larger size than exosomes 100–1000 nm and apoptotic bodies which are released from apoptotic cells and are 1 μm–5 μm in size [19]. Therefore, studies looking at the selective release of microRNAs have focused on the exosomal fraction of extracellular vesicles. It must be noted that it is difficult to achieve pure isolates of exosomes experimentally, meaning that most studies report RNA data from enriched populations of exosomes which still contain protein bound microRNAs or other extracellular vesicles [14], [15]. Studies have sought to examine if circulating microRNAs are mainly protein bound or enclosed in extracellular vesicles. Turnchovich et al. [12] found that the majority of circulating microRNAs are associated with AGO2 protein and only a small proportion of circulating microRNAs are enclosed in extracellular vesicles. They hypothesised that microRNA/AGO2 complexes are released by cells during apoptosis [12]. A global profiling study has suggested that AGO2 may play a role in a selective pathway, which targets microRNAs into extracellular vesicles [20]. This study compared different cell lines and identified a subset of microRNAs that are released in extracellular vesicles through a common mechanism for sorting microRNA [20]. These microRNAs are not processed through the canonical pathway by Dicer, but through an AGO2 mediated maturation pathway, which leads to preferential release in extracellular vesicles [20].

## 3 Exosomal RNA Function

Several types of RNA have been found in exosomal fractions, with microRNAs and mRNAs being the most abundant species. These microRNAs and mRNAs were found to be enclosed inside exosomes, as opposed to being attached to the outside. Exosomes isolated from cultures of glioblastoma cells were found to fuse with the membranes of host cells and release the contents into the host cell [21]. The exosomal contents were found to be fully functional in recipient cells, with microRNAs promoting the downregulation of their targets [21] and exosomal mRNAs being translated into protein in the host cell [22]. In contrast, a study by Chevillet et al. [23] questions the physiological relevance of exosomal microRNA, as stoichiometric analysis of the absolute copy number of microRNAs and the number of exosomes indicates that even for abundant cancer biomarkers (in this case prostate cancer) there is less than one microRNA copy per exosome. Therefore, this study brings into question whether or not exosomal microRNAs are in sufficient abundance in exosomes to have an effect on the recipient cells. Potentially, a high level of exosome uptake or non-canonical microRNA activation would be required to elicit exosomal microRNA-mediated effects [23]. However, given that numerous exosomal microRNAs have been shown to have functional effects (discussed below), more research is needed to ascertain the stoichiometric requirements for exosomal microRNA function.

## 4 Passive Release of microRNAs from Cells

Several studies have found that the bulk of microRNA released in exosomes reflects the cellular microRNA expression profile [24], [25], and the majority of microRNAs (~ 66%) are released from cells passively by mass action. miR-16 was identified as a microRNA that represents this passive exosomal microRNA release, and the amount of miR-16 in the cell matches the amount found in exosomes [25]. In agreement with this idea, Squadrito et al. [26] demonstrated that the microRNA content of exosomes is determined by the levels of the microRNA target mRNA in the cell. For example, if there is an abundance of the mRNA target of the microRNA, the microRNA is bound and this decreases the amount of that specific microRNA in the exosomes. If there is a high abundance of the microRNA relative to its targets, then an increased level of the microRNA is observed in the exosomes, leading to the suggestion that the exosome pathway is a mechanism to maintain microRNA:mRNA homeostasis in the cell [26]. In contrast to this passive release mechanism, a subset of microRNAs are overrepresented in exosomes, meaning that they are enriched in comparison to levels observed in the cell, indicating a selective release mechanism [25].

## 5 Selective Release of microRNAs from Cells

Investigation of the mechanistic requirements governing microRNA inclusion in to exosomes found that the neutral sphingomyelinase 2 (nSMase2) is required for microRNA exosomal release [27]. The nSMase2 enzyme catalyses the rate limiting step in ceramide synthesis. Ceramide is required to promote budding in the endosomal compartment. Therefore, inhibition of nSMAse2 inhibits the formation of exosomes [27], [28]. The active selection of microRNAs for packaging into exosomes has been suggested to be sequence specific [29], with the selection of microRNAs for inclusion into exosomes being determined through the binding of chaperone proteins. The protein hnRNPA2B1 has been shown to play a key role in targeting a subset of microRNAs with a specific motif into exosomes [30]. In addition, 3′ modifications of microRNAs have been suggested to determine whether a microRNA is retained in the cell or exported in exosomes. Indeed, non-templated additions of 1,2 or 3 bases of uridine or adenosine to the 3′ end of certain microRNAs has been found to influence microRNA release into exosomes, with poly-adenylated microRNAs being more likely to be retained in the cell and poly-uridynilated microRNAs packaged into exosomes [31].

Alterations in cancer-associated cell signalling pathways also alter the microRNA profile of exosomes [32], [33]. For instance, p53 has been shown to govern the release of exosomes from cells. On activation by DNA damage, p53 transcriptionally up-regulates the expression of tumour suppressor activated pathway 6 (TSAP6), which has been found to be essential for p53-mediated exosome release [34]. Although a direct mechanism linking changes in microRNA content in these exosomes has not yet been described, it illustrates the influence that cell signalling pathways have on exosomal release. In colorectal cancer cells, exosomal loading has been shown to be dependent on KRAS mutational status, with mutant KRAS cells releasing higher levels of miR-100 and wild-type KRAS cells releasing higher levels of miR-10 into the exosomal fractions [26], [35], through a mechanism requiring nSMAse. Additionally, KRAS-dependent activation of MEK-ERK signalling inhibits sorting of AGO2 bound microRNAs into exosomes [36]. Although it is clear that oncogenic signalling pathways influence microRNA content in exosomes, the exact mechanisms by which these microRNAs are sorted into exosomes and the contribution of passive versus active sorting remain poorly defined. These studies underline the need for further research to determine the contribution of passive and selective release of microRNAs from cancer cells.

## 6 Extracellular microRNAs in the Tumour Microenvironment

**Fig. 1:**
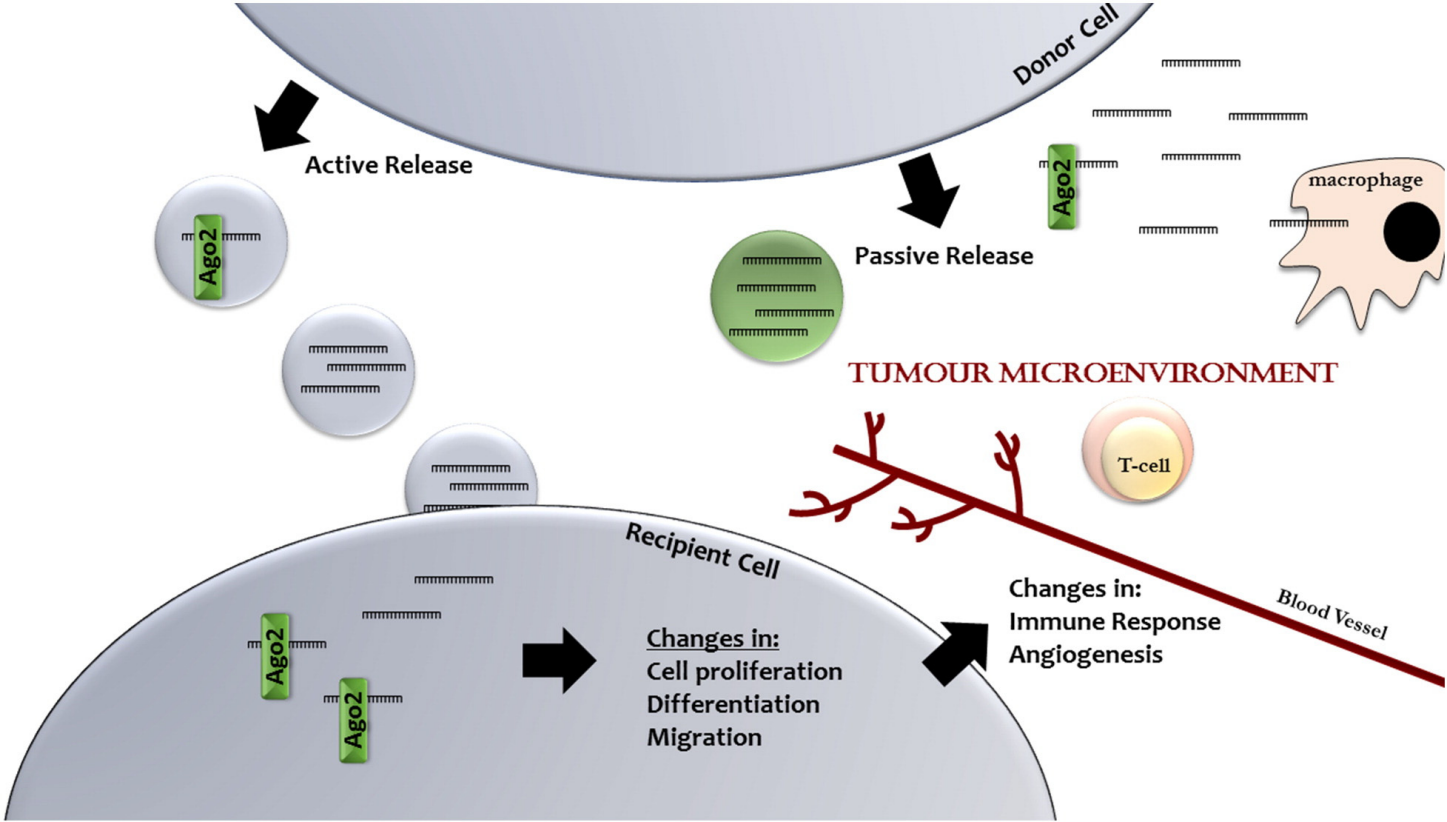
Cell-free microRNAs are taken up by cells in the tumour microenvironment. MicroRNAs are secreted by donor cells through active or passive release mechanisms. These cell-free microRNAs are then taken up by recipient tumour, immune, or stromal recipient cells present in the TME, where they elicit functional effects on gene expression in recipient cells leading to changes in cell proliferation, differentiation, migration, immune response, and angiogenesis that collectively influence tumour progression and metastasis.

MicroRNAs expressed within tumour cells, fibroblasts, immune cells, and endothelial cells present in the TME are known to promote tumour growth and metastasis by targeting mRNAs in their cell of origin, leading to changes in cellular phenotype and changes in cytokine expression and secretion [4], [37]. Emerging evidence indicates that in addition to this canonical intracellular mechanism, cell secreted microRNAs can be delivered to other cells in the tumour microenvironment leading to reprogramming of the target cell transcriptome and altering tumour growth, angiogenesis, metastasis and immune function in a paracrine manner (Fig. 1, Table 1).

### 6.1 Exosomal microRNA in Cell Fate Determination and Angiogenesis

Stem-cells are hypothesised to influence the cellular differentiation of other cells through secretion of growth factors, cytokines, and exosomes. Indeed, mesenchymal stem cell (MSC) - derived exosomes isolated from mice with pulmonary hypertension have been shown to induce pulmonary hypertension in healthy mice [38]. However, the exact mechanisms through which stem-cell derived exosomes exert these effects remains unknown. MSCs are recruited to the TME where they can differentiate into tumour-associated fibroblasts and produce ECM matrix proteins necessary for the development of a tumour promoting microenvironment [39]. Crosstalk between MSCs and tumour cells that occurs through the release of exosomal microRNAs has been shown to have a key role in either suppressing or driving angiogenesis in tumours. For example, exosomal miR-16 released by MSCs can suppress angiogenesis by down-regulating VEGF expression in breast cancer cells [40]. The miR-17-92 cluster contains some of the most well characterised oncogenic microRNAs and is known to target cell cycle inhibitors CDKN1A and CDKN1C and E2Fs [41], [42], [43]. Significant for tumourigenesis, the miR-17-92 cluster promotes angiogenesis by targeting the anti-angiogenic factors connective tissue growth factor, thrombospondin-1, and integrin alpha5 [44], [45]. In addition to this intracellular signalling mechanism, members of this cluster of microRNAs have been found to be exported from cells in exosomes [46], with more recent evidence indicating that these exosomal miRs originating from tumour cells can elicit changes in the surrounding TME. Interestingly, miR-92a has been shown to have both pro-angiogenic and anti-angiogenic roles depending on which cell type it is secreted from. When released in tumour exosomes it plays a pro-angiogenic role, whereas when released by MSCs it exhibits an anti-angiogenic role [46], [47]. Exosomal microRNAs released from tumour cells have also been shown to influence angiogenesis by effecting MSCs and other cells in the TME. Exosomal microRNAs released by chronic lymphocytic leukaemia cells are taken up by both endothelial and mesenchymal stem cells, altering the transcriptome of stromal cells and leading to the release of pro-angiogenic factors [48]. Additionally, transformed lung cancer cells have been shown to transfer miR-21 via exosomes to nearby normal bronchial epithelial (HBE) cells. This increases VEGF production and promotes angiogenesis through a STAT3 dependent mechanism [49]. Similarly, hypoxic conditions induce A549 lung cancer cells to release miR-494 containing exosomes to surrounding endothelial cells, which leads to the suppression of PTEN and Akt/eNOS pathway activation, enhancing angiogenesis. Treatment of these tumours with anti-miR-494 has been shown to inhibit angiogenesis and attenuate the growth of tumour xenografts in this model [50].

### 6.2 Exosomal microRNA in the Promotion of Tumour Cell Migration and Metastasis

The complex process of metastasis involves multiple steps including local invasion, intravasation, and survival in the vascular system, extravasation, and colonisation of distant organ sites. These steps are often highly dependent on interactions between the tumour cell and the local microenvironment [51]. A key step in this process is for cancer cells to acquire a certain degree of plasticity to adapt to their new environment. The miR-200 family, which contains miRs-200a,-200b,-200c, -141 and -429, was traditionally thought to encode tumour suppressor microRNAs, due to its ability to downregulate Zeb1 expression and suppress epithelial to mesenchymal transition (EMT). However, Le M.T. N et al. recently showed that metastatic capability could be transferred between metastatic and non-metastatic cells via extracellular vesicles containing miR-200. MiR-200 containing extracellular vesicles taken from metastatic breast cancer cells could confer the ability to colonise distant tissues on weakly metastatic breast cancer cells by altering gene expression profiles and promoting mesenchymal-to-epithelial transition [52]. A similar phenomenon has been observed in metastatic breast cancer cells where the secretion of miR-10b from metastatic breast cancer cells has been shown to induce invasive properties on non-malignant cells [53]. Additionally, miR-21 containing exosomes have been shown to promote migration and invasion of recipient esophageal cancer cells by targeting programmed cell death 4 (PDCD4) and activating the c-Jun N-terminal kinase (JNK) pathway [54]. These studies highlight the role that extracellular microRNAs play in tumour cell-cell communications and mediating plasticity changes within tumour cells to promote a metastatic phenotype. In addition to modulating nearby tumour cells, secreted microRNAs can also act on other cells in the TME to enhance metastasis. For example, miR-105 is secreted by metastatic breast cancer cells promotes vascular permeability by downregulating the tight junction protein ZO-1 in endothelial cells. Inhibition of miR-105 in highly metastatic tumours led to a reduction in tumour invasiveness and a restoration of vascular barrier function, thereby decreasing metastasis [55]. Taken together, these studies illustrate that exosomal microRNAs secreted by tumour cells are capable of promoting metastasis by inducing invasive properties in nearby tumour cells and by manipulating the tumour microenvironment to enhance tumour cell migration.

### 6.3 Exosomal microRNA and Modulation of Tumour Immune Response

Tumour progression is intricately linked to the immune response. Activation of specific immune cells such as dendritic cells (DCs), natural killer cells (NK cells), and CD8 + effector T cells drive potent anti-tumour responses, while suppression of these cell types promotes tumour survival. Cancers are known to subvert the functions of other cells in the tumour microenvironment, limiting local immune responses and ultimately leading to enhanced invasion and tumour progression [56]. Secretion of exosomal microRNAs from various cell types present in the tumour microenvironment may play a role in modulation of immune response in the tumour microenvironment. Indeed, exosomal miR-9 secreted by tumour cells has been shown to inhibit expression of major histocompatibility complex (MHC) class I, thereby preventing recognition of tumour cells by the immune system [57]. Macrophages are key regulators of host immunity, and tumour-associated macrophages (TAMs) exert immunosuppressive effects through the secretion of cytokines that suppress anti-tumour responses. It has been demonstrated by Fabbri M. et al. [58] that exosome-derived microRNAs bind to human Toll-like receptor 8 (TLR8), which shares the same function as murine TLR7. Exosome-derived microRNA binding to these receptors stimulates downstream NFκB pathway activation and the secretion of inflammatory cytokines in macrophages [58]. Along these same lines, binding of exosome-derived miR-21 to TLRs on macrophages has been shown to induce the secretion of inflammatory cytokines and the promotion of metastasis [58]. Additionally, IL-4 activated macrophages have been found to secrete exosomes containing miR-223, which promotes invasion of breast cancer cells [59]. Interestingly, the same secreted microRNA can have differential effects on the microenvironment depending on what cell type it acts on. For example, when exosomal miR-21 binds to TLR7/8 on macrophages, it promotes secretion of IL-6, leading to a pro-inflammatory response. However, when miR-21 binds to TLR7 receptors on myoblasts, it promotes cell-death through activation of the JNK pathway [60]. DCs are antigen presenting cells (APCs) that play an important role in antitumor response by facilitating the activation of T-cells in the tumour microenvironment. Pancreatic cancer cells have been shown to repress DC activation by releasing exosomes containing miR-203 which is then taken up by DCs resulting in downregulation of TLR4 expression and decreased production of cytokines TNF-α and IL-12 [61]. Additionally, miR-23 containing exosomes secreted by hypoxic tumour cells are taken up by NK cells leading to the suppression of CD107a and leading to immunosuppression [62]. Exosomally-derived microRNAs have also been shown to modulate T-cells present in the tumour microenvironment. For instance, exosomal microRNAs (miR-24-3p, miR-891a, miR-106a-5p, miR-20a-5p, and miR-1908) secreted by nasopharyngeal carcinoma (NPC) cells have been shown to promote regulatory T-cell (Treg) generation leading to suppression of anti-tumour immunity [63]. Tregs themselves have also been shown to exert some of their immune suppressive effects through exosomal microRNA-mediated effects. Indeed, microRNAs released in Treg derived exosomes were shown to inhibit Ptgs2, thereby limiting Th1 responses [64]. It is clear that microRNAs can impact gene expression in immune cells. However, the implications of the bi-directional exchange of microRNA between tumour and immune cells is only beginning to be uncovered. Given that microRNAs are only one component of the cargo contained within exosomes, it will be important to gain a better understanding of what extent they contribute to exosome-induced changes in the TME. Moreover, more research is needed to characterise the microRNAs expressed and secreted from each of the different immune cell types and to what extent this effects tumour progression.

## 7 Conclusions and Future Prospects

It is well established that microRNAs play essential roles in modulating the intracellular signalling pathways in tumour cells and other cells present in the TME. Now, emerging data indicates that these processes are even more complex, with the bi-directional exchange of microRNAs occurring between various cell types present in the TME. The process of microRNA release occurs both passively and actively, although clearly more research to define the exact mechanisms through which microRNAs are sorted for extracellular release is needed. Further investigation and understanding of microRNA release mechanisms, as well as the stoichiometry required for functional effects are needed to gain a better understanding of the mechanisms governing downstream effects. Moreover, very little is known about the mechanistic requirements for microRNA uptake by target cells. Broader understanding of these processes would greatly contribute to understanding the complexity of the TME that drives tumour progression and could open new opportunities for therapeutic intervention or new biomarkers for the treatment of cancer.

## Conflict of Interest

E. Bell and M.A. Taylor are employees of AstraZeneca.

## Figures and Tables

**Table 1:** 

MicroRNA	Cell type released by	Cell type taken up by	Extracellular Role	Ref.
*Angiogenesis*				
miR-16	MSCs	Tumour cells	Suppression of angiogenesis by downregulating VEGF.	[40]
miR-92a	Tumour cells MSCs	MSC Endothelial cells	Pro-angiogenic role Anti-angiogenic role.	[46], [47]
miR-21	Tumour cells	Bronchial epithelial cells	Increase in VEGF production and promotion of angiogenesis	[49]
miR-494	Tumour cells	Endothelial cells	Suppression of PTEN and Akt/eNos pathway activation, enhancing angiogenesis.	[50]

*Migration and metastasis*				
miR-200	Metastatic tumour cells	Non-metastatic tumour cells	Promoting mesenchymal-to-epithelial transition and colonisation of metastatic sites.	[52]
miR-10b	Metastatic tumour cells	Non-malignant cells	Induction of invasive properties	[53]
miR-21	Tumour cells	Tumour cells	Promoting migration and invasion by targeting PDCD4 for downregulation.	[54]
miR-105	Metastatic tumour cells	Endothelial cells	Promoting vascular permeability by downregulating ZO-1.	[55]

*Modulation of tumour-immune response*				
miR-9	Tumour cells	Tumour cells	Inhibits expression of MHC class I, preventing recognition of tumour cells by the immune system.	[57]
miR-21	Tumour cells	Macrophages Myoblasts	Binds to TLRs, inducing the secretion of inflammatory cytokines. Induction of cell-death through activation of the JNK pathway.	[58], [60]
miR-223	Macrophages	Tumour cells	Promotion of tumour cell invasion.	[59]
miR-203	Tumour cells	Dendritic cells	Downregulation of TLR4 expression and decreased production of cytokines TNF-α and IL-12.	[61]
miR-23	Tumour cells	NK cells	Downregulation of CD107a leading to immunosuppression.	[62]
miR-24-3p, miR-891a, miR-106a-5p, miR-20a-5p, miR-1908	Tumour cells	T-cells	Promotes Treg differentiation leading to suppression of anti-tumour immunity.	[63]
